# Regulation of the Complement System by Pentraxins

**DOI:** 10.3389/fimmu.2019.01750

**Published:** 2019-08-02

**Authors:** Karita Haapasalo, Seppo Meri

**Affiliations:** ^1^Department of Bacteriology and Immunology and Translational Immunology Research Program, University of Helsinki, Helsinki, Finland; ^2^HUSLAB, Helsinki University Hospital, Helsinki, Finland; ^3^Department of Biomedical Sciences, Humanitas University, Milan, Italy

**Keywords:** CRP–C-reactive protein, complement factor H, PTX3, innate, age-related macular degeneration (AMD), factor H-related protein, complement C1q, cholesterol

## Abstract

The functions of pentraxins, like C-reactive protein (CRP), serum amyloid protein P (SAP) and pentraxin-3 (PTX3), are to coordinate spatially and temporally targeted clearance of injured tissue components, to protect against infections and to regulate related inflammation together with the complement system. For this, pentraxins have a dual relationship with the complement system. Initially, after a focused binding to their targets, e.g., exposed phospholipids or cholesterol in the injured tissue area, or microbial components, the pentraxins activate complement by binding its first component C1q. However, the emerging inflammation needs to be limited to the target area. Therefore, pentraxins inhibit complement at the C3b stage to prevent excessive damage. The complement inhibitory functions of pentraxins are based on their ability to interact with complement inhibitors C4bp or factor H (FH). C4bp binds to SAP, while FH binds to both CRP and PTX3. FH promotes opsonophagocytosis through inactivation of C3b to iC3b, and inhibits AP activity thus preventing formation of the C5a anaphylatoxin and the complement membrane attack complex (MAC). Monitoring CRP levels gives important clinical information about the extent of tissue damage and severity of infections. CRP is a valuable marker for distinguishing bacterial infections from viral infections. Disturbances in the functions and interactions of pentraxins and complement are also involved in a number of human diseases. This review will summarize what is currently known about the FH family proteins and pentraxins that interact with FH. Furthermore, we will discuss diseases, where interactions between these molecules may play a role.

## Introduction

As a part of the host defense, the immune system enables us to cope with unwanted materials threatening our body. Innate immunity acts stereotypically and rapidly (in minutes to hours) to recognize and clear away unwanted materials, while the adaptive immunity generates antigen-specific responses during a longer time course (days to weeks). The central players in the humoral arm of innate immunity include complement (C) system components and soluble pattern recognition molecules, such as pentraxins and collectins. The interplay between these components has a crucial role in the recognition and clearance of both foreign and endogenous unwanted particles from the human body. Any disturbances in these interactions may have a significant impact on the immune response and health.

The complement system was first described in 1888–1889, when both George Nuttall and Hans Buchner independently demonstrated that blood serum was able to kill bacteria. Buchner called this activity “alexin.” However, due to the “complementing” function, 10 years later the system was named “complement” by Paul Ehrlich. Jules Bordet observed that for bacterial killing serum contains a heat-stable component, i.e., antibodies, and a heat-labile component, complement. Within the next 50 years it was generally believed that complement requires antibodies for activation. In 1954, however, Louis Pillemer demonstrated that the complement system can be activated independently from antibodies by the so called “properdin” system, thereby playing a central role in innate immunity (1). Because this pathway does not require antibodies nor humoral lectins for activation, like the classical (CP) and lectin pathways (LP), it was later named as the alternative pathway (AP). The AP can act as a separate pathway and as an amplification system of activation triggered by the other pathways. We now know that the first identified heat-labile components, C1 subcomponents C1r and C1s, belong to an activation cascade containing over 40 different molecules. The heat-labile components include also other serine proteases of the C system, like C2, factor B, and the lectin-associated serine proteases (MASPs). Many of the complement factors also interact e.g., with the coagulation, fibrinolytic, and kinin system components. Complement also closely links the innate and adaptive immune systems together e.g., in antigen recognition and delivery to the adaptive immune system players: dendritic cells, follicular dendritic cells, macrophages, B cells and T cells (2). Importantly, the immune system also maintains tolerance and controls excessive inflammatory reactions.

A unique and separate system of targeted complement activation involves a group of evolutionarily relatively old molecules, the pentraxins. C-reactive protein (CRP), serum amyloid P component (SAP), and pentraxin-3 (PTX3) belong to the pentraxin family of pattern recognition molecules. The listed three members have been shown to interact with distinct C components. The first interaction between the C component C1q and CRP was described by Volanakis and Narkates (3). Thereafter, an interaction between SAP and C1q was soon reported (4). Years later, PTX3 was found to bind C1q, as well (5). These data and further studies have shown that pentraxins play a crucial role in inflammation in directing C activation toward, for example, foreign microbes, apoptotic cells and injured tissue. They interact with C components at different stages of the activation cascade. It has been generally accepted that, together with the C system, they contribute to host defense, tissue clearance and regulation of inflammation.

In addition, but very importantly, after activating the complement classical pathway the pentraxins regulate further activation to prevent excessive tissue damage and to coordinate targeted clearance of the injured tissue components. The complement inhibitory function of pentraxins is partially based on their ability to interact with factor H (FH), a complement regulator that interferes with AP activity at the C3b stage and thus prevents formation of the complement membrane attack complex C5b-9 (MAC). Pentraxins and C components such as C3b, C5b-9, and FH are often found in pathological deposits. Changes in their temporal behavior correlate and associate with the same diseases (6–8). Mutations or polymorphisms in these molecules can influence the interactions and have an impact on the progression of the diseases (6–8). The roles of FH, pentraxins and the interactions between these molecules during the course of inflammation have been the subject of many investigations. Pentraxins have been considered either as inflammatory or as anti-inflammatory factors. Thus, their potential causal or protective roles in various diseases still remain to be sorted out. This review summarizes studies on the interactions between pentraxins and the complement system, We will highlight current observations and discuss aspects, where more research is needed.

## The Complement System

**Figure 1:**
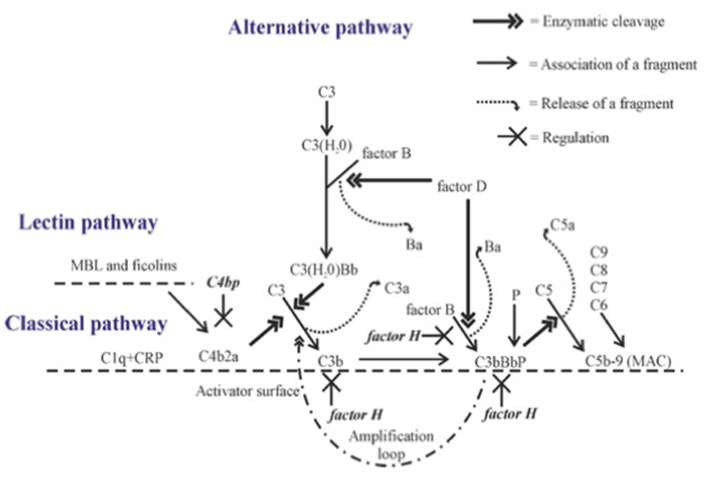
Complement activation with emphasis on alternative pathway amplification. Complement proteins interact with each other in sequence leading to cleavage of C3 to C3b. Activation on a suitable target leads to opsonization (coating with C1q, C4b, C3b, or iC3b), release of chemotactic and anaphylatoxic fragments (C5a, C3a) and formation of the membrane attack complex (MAC). C4bp inhibits the CP C3 convertase C4b2a. The alternative pathway gets amplified, when C3 convertase (C3bBb) activates additional C3 molecules by cleavage to C3b to generate new C3 convertase enzymes. This amplification step efficiently opsonizes the target with C3b molecules and its inactivation fragment iC3b. Factor H is the main inhibitor of the amplification loop. Its function is to promote C3b inactivation, inhibit binding of factor B to C3b and accelerate the dissociation of the AP C3 convertases.

The complement cascade can be activated through three pathways, the classical, lectin and alternative pathways (Figure 1). C3 is the key component of all three pathways, since all pathways converge on it, and major effector functions of complement are mediated through activation of this molecule.

### Alternative Pathway

Distinct from the CP and LP, the AP is activated spontaneously, because C3 is continuously hydrolyzed at a low rate in human plasma to form a metastable C3(H_2_0) without cleavage of C3 to C3a and C3b (Figure 1). C3(H_2_0) is able to bind factor B in a Mg^2+^-dependent manner exposing it to cleavage by factor D thus forming the C3(H_2_0)Bb complex, the initial C3 convertase, in the fluid phase. This enzyme cleaves fluid phase C3 to C3a and C3b, and the freshly formed C3b can then target any nearby surface that has available hydroxyl or amino groups for covalent attachment. Soluble or fluid phase associated C3bBb enzyme has a strong catalytic activity for cleaving new C3 molecules to C3a and C3b and thus to amplify AP activation. The smaller cleavage fragment, C3a, is released into solution and acts as an anaphylatoxin and as a chemotactic and activating factor for leukocytes (2).

A key to the properly directed and efficient complement attack by AP is the ability to discriminate the target cells from host cells. In general, on the host cell surface the C3b molecules are rapidly inactivated, while on foreign cells and particles the deposited C3b molecules remain active and can lead to rapid amplification of AP activation. The C3 convertases (C3bBb) also activate the terminal complement cascade by cleaving fluid phase C5. Additional nearby C3b molecules may be needed for the attraction and proper orientation of C5 molecules. C5 activation leads to the release of the strongly proinflammatory chemotactic and anaphylatoxic protein fragment C5a and assembly of the potentially lytic C5b-9 membrane attack complex (MAC) onto the target membrane. Therefore, the fate of C3b deposits on a cell membrane dictates whether complement activation eliminates the target or not. Because of the strong biological activities of the C system, its activation needs carefully directed and efficient regulation at different times, occasions and locations. For this, additional molecules like the pentraxins are needed.

## Factor H and Factor H-Related Proteins

The molecular mechanism, how our own cells are protected from the AP attack, is based on the recognition of C3b on host cells by factor H (FH), the main AP regulator in plasma and other body fluids. FH is an elongated molecule composed of 20 short-consensus repeat (SCR) or complement control protein (CCP) domains. The N-terminal domains 1-4 are responsible for the regulatory activity, while the C-terminal domains 19-20 are responsible for simultaneous recognition of C3b (9) and either sialic acids or glycosaminoglycans present on self surfaces (10). In addition, domains 6-7 can bind to surface polyanions (11, 12), As a result of these interactions, FH blocks AP activation and amplification on host structures. FH does this by acting (i) as a cofactor for factor I in the proteolytic cleavage of C3b to iC3b, (ii) by inhibiting the formation or (iii) by promoting the decay of the surface-bound C3bBb convertases (Figure 1) (13–15).

The essential role of FH in keeping spontaneous complement activation in check is obvious. It is based on the clinical consequences of *CFH* gene mutations or anti-FH autoantibodies that prevent full function of FH (16–18). Although the initiation of AP activation in the fluid phase relies on a spontaneous low-grade process without a need for a trigger, the activation will be enhanced under suitable conditions. Disease-related FH abnormalities usually lead to an imbalance between AP activation and regulation in the fluid phase or to a mistargeted attack against endothelial and blood cell surfaces (19). On surfaces recognized as activators AP amplification readily takes place, because the generated C3b molecules can bind covalently to the surface in the immediate neighborhood of the activating C3 convertase.

**Figure 2:**
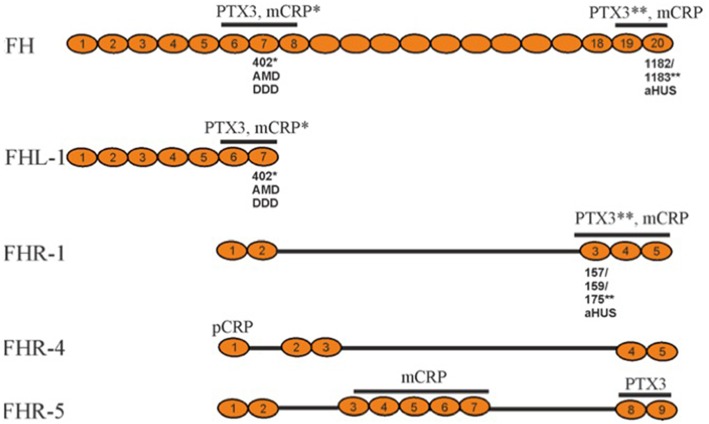
Schematic structures of representative factor H family proteins, their interactions with pentraxins CRP and PTX3 and disease associations. The pentraxin interacting domains in FH family proteins (marked above) display disease-associated polymorphisms that alter the protein/pentraxin interactions. The substituted amino acid are marked below. The asterisk indicates the pentraxin, whose binding to the protein is affected by the polymorphism. FHR-2 and FHR-3 are not shown, because their plasma concentrations are low. AMD, Age-related Macular Degeneration; DDD, Dense Deposit Disease; aHUS, atypical Hemolytic Uremic Syndrome; PTX, pentraxin; CRP, C-reactive protein; mCRP, monomeric CRP; pCRP, pentameric CRP.

In addition to FH, the factor H family includes an alternatively spliced variant of FH, called factor H-like protein (FHL-1), and five factor H-related proteins (FHR-1 to 5) (Figure 2). While FHL-1 contains the first seven domains of FH (plus an extra 4 unique amino acids) and possesses AP regulatory activity, FHRs in general lack these regulatory domains. Therefore, FHRs have no strong direct regulatory activity, although they all interact with C3b (20). Instead, they can compete with the binding of the C-terminus of factor H and thereby regulate its activity with a net result to promote complement activation (21). The most homologous regions between FH and the FHRs are the 2 most C-terminal regions (19-20 in FH), which bind to the C3d region of C3b (22).

The gene cluster coding for FH and FHR-proteins is located on chromosome 1q32. The full-length FH is encoded by 22 exons, while the sequence for FHL-1 stops after alternative splicing at exon 10. The *CFHR* genes are located downstream from the *CFH* gene (23).

There are several known genetic variations and mutations within the *FH* gene cluster. Of these, some have no observable effect on the phenotype, while others are associated with diseases or other harmful effects on the carrier. Most of the disease-related mutations in FH are located within the carboxyl-terminal domains 19-20. They are associated with the atypical hemolytic-uremic syndrome (aHUS) (24). Mutations in the amino-terminus are associated with dense deposit disease (DDD), earlier called membranoproliferative glomerulonephritis type II (MPGN) and rarely also with partial lipodystrophy (PLD). Some polymorphisms have been found to be associated with age-related macular degeneration (AMD), which is the most common cause of visual loss in the elderly people in the industrialized countries. The strongest genetic risk factor for AMD is the Y402H (Tyr402His) polymorphism, which is located in the domain seven (CCP7) of FH (25–27). In addition to polymorphisms and mutations, also autoantibodies against FH can predispose to diseases similar to aHUS or DDD (28, 29). Individuals with factor H deficiency have an over 1,000-fold increased risk to develop meningococcal meningitis, which is due to a secondary C3 and C5 deficiency following overactivation of the alternative pathway in the fluid phase.

## FH Interactions With Pentraxins

### Pentraxins

**Figure 3:**
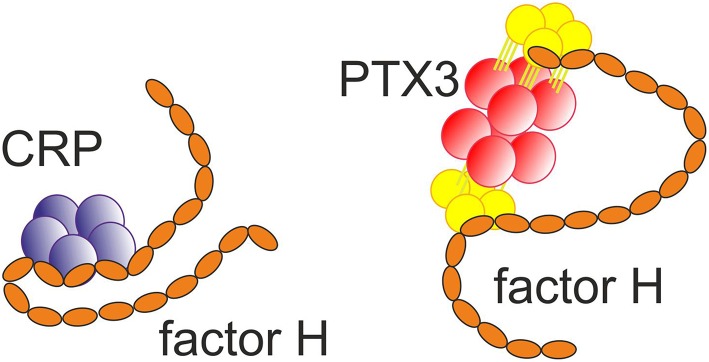
Binding of the short pentraxin CRP (32) and the long pentraxin PTX3 (33) to factor H. Multiple interactions between the molecules exist.

Pentraxins (PTX) are innate pattern recognition molecules, some of which are produced as a response to infection and tissue damage. The name pentraxin comes from the ability of at least some of these molecules to form multimers with five nearly identical subunits. Pentraxins have multiple functions. The best characterized function is activation of the classical pathway of complement on certain microbes and necrotic cells, and thereby contribution to removal of cellular debris. Further observations also imply antibody-like functions, which in evolution would predate the emergence of adaptive immunity (30). The pentraxins are divided into two groups, the short pentraxins: C-reactive protein (CRP) and serum amyloid P component (SAP) and long pentraxins: neuronal PTX1 (NPTX1), neuronal PTX2 (NPTX2), PTX3 and PTX4. All PTXs contain an approximately 200 amino acid-long PTX domain, while the long PTXs have an additional N-terminal domain. The neuronal pentraxins, NPTX1 and NPTX2, are expressed particularly, but not exclusively, in neurons, They have been suggested to be involved in the clearance of synaptic debris during neuronal synapse remodeling (31). However, no role in complement activation by these molecules has been reported. In contrast, CRP, SAP, and PTX3 are all known to activate complement, interact with multiple complement components and thereby contribute to innate immunity. Sometimes, they have been referred to as ancestors of antibodies (Figure 3).

SAP shares approximately 51% sequence identity with CRP, which supports the hypothesis that SAP and CRP are products of an earlier gene duplication event. SAP is the glycoprotein precursor of the amyloid P protein. SAP occurs in association with amyloid deposits, including those associated with Alzheimer's disease (34). SAP binds C1q to activate the CP similarly as CRP and PTX3. However, according to the current knowledge, SAP does not interact with any of the FH family proteins. Instead, SAP binds the fluid phase regulator of the CP, C4b-binding protein (C4bp), and plays a potential role in the regulation of CP (35).

CRP was originally named by its ability to bind to the phosphocholine (PC) part of the C-type polysaccharide of pneumococcus in a calcium-dependent manner. It also binds on carbohydrate structures of many other microorganisms such as fungi, yeasts, bacteria and parasites. Moreover, it recognizes modified low-density lipoproteins (LDL) and necrotic and apoptotic cells, and thereby participates in the phagocytosis and clearance mechanisms of the innate immune response (36). One of the specific targets for CRP in LDL particles is cholesterol itself, to which CRP binding was found to be dependent on the 3beta-OH group (37). CRP is produced by hepatocytes as a response to infection or tissue damage, mainly in response to the proinflammatory cytokine IL-6. CRP is therefore commonly used as a non-specific laboratory indicator for infection, systemic inflammation, and tissue damage (34). Highly elevated levels are usually seen in serious bacterial infections, but not so commonly in viral infections. Binding of CRP to apoptotic and necrotic cells enhances their opsonization and phagocytosis by macrophages.

**Figure 4:**
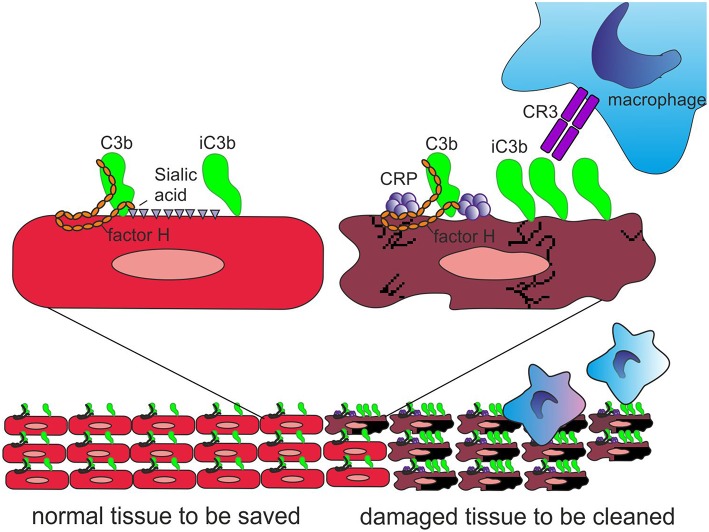
Role of CRP and FH in promoting clearance of dying cells. On viable cells **(Left)** accidentally deposited C3b is rapidly inactivated to iC3b and no C activation takes place. On surfaces, from where protective polyanions, like sialic acids, are lost as a consequence of cell damage, factor H binding is decreased **(Right)**. This together with CRP-mediated classical pathway activation leads to complement activation. During a prolonged time course, the large number of deposited C3b molecules, will, however, be inactivated to iC3b with the help of factor H recruitment by CRP. Deposited iC3b molecules promote phagocytosis of the debris without leading to further activation of the terminal pathway.

Importantly, CRP has been observed to bind the alternative complement pathway inhibitor factor H (FH) to potentially recruit it to areas of tissue damage (32). This would limit AP activation and excessive inflammation in these areas and promote a non-inflammatory clearance of dying cells (38) (Figure 4). With the help of complement, CRP thus demarcates the area destined to clearance.

In addition to FH, also FHR-1, FHR-4, and FHR-5 (Figure 2), have been shown to bind CRP on necrotic cells (39–41). When compared to full length FH and FHL-1, the FHRs, however (with the possible exception of FHR-5), possess no direct complement regulatory activity. It has been suggested that FH, FHL-1, and different FHRs possess different binding properties to CRP than FH. FHL-1 domains 6-7, FHR-1 domains 3-5, and FHR-5 domains 3-7 preferentially interact with the monomeric (mCRP), while FHR-4 domain 1 mainly binds the pentameric form of CRP (pCRP) (39, 41–43). Both CRP forms are known to exist in humans. They have been shown to possess similar functions in modulating CP activation on necrotic cells, but they differ in their relative abundance in different tissues. The pCRP is present in plasma, while the mCRP is detected mainly on the surfaces of damaged cells and platelets (44). While the molecular function of the FH-CRP interaction is known, it is still unclear whether binding of FHRs to CRP will enhance C activation and/or promote CRP-mediated opsonization. The FHRs, however, appear to play a particular role in C activation, as exemplified by the association of several reported genetic variations, e.g., FHR deletions and hybrid molecules, with various diseases (45).

Unlike CRP and SAP, PTX3 has been described as an octamer composed of eight identical subunits. It is produced locally in a number of tissues and expressed by several cell types, including fibroblasts, monocytes, macrophages, myeloid dendritic cells and neutrophils (3). It can opsonize target surfaces, such as fungal (*Aspergillus*) and bacterial pathogens and apoptotic cells to initiate complement activation. PTX3 binds C1q, mannan-binding lectin, M-ficolin (ficolin-1) and L-ficolin (ficolin-2), and thereby activates both the CP and LP (36). Binding of PTX3 to C1q is calcium-independent, as opposed to CRP and SAP that both require this divalent cation for their interaction with C1q (34). In addition, PTX3 binds FH, and FHL-1 to inhibit excessive complement activation (27). Also, FHR-1 and FHR-5 have been observed to bind PTX3. By competing out factor H FHRs may actually promote complement activation.

## Alterations in FH-Pentraxin Interactions and Their Possible Disease Associations

Recently, it has become clear that AP dysregulation is a central event in the development of several complement related-diseases involving factor H mutations or polymorphisms in domains FH1-5, FH7, and FH19-20 (Table 1). While mutations in FH19-20, or autoantibodies against this region, are associated with atypical hemolytic uremic syndrome (aHUS), the Y402H polymorphism in domain 7 is associated with age-related macular degeneration (AMD) (54, 55) and dense deposit disease (DDD) or C3-glomerulonephritis (C3GN) (26). DDD and C3GN are collectively referred to as C3 glomerulopathy (C3G), which is linked to mutations in the N-terminus of FH or to FHR abnormalities. Interestingly, the AMD/DDD-associated domain 7 of FH mediates binding to CRP as well as to glycosaminoglycans (56). In addition to this short pentraxin, the long pentraxin 3 (PTX3) interacts with FH (33). However, unlike CRP binding to FH, the PTX3 binding to FH is not affected by the AMD-associated polymorphism. This implies different molecular functions for these two pentraxins within the complement regulatory system. Because CRP and PTX3 are both acute phase proteins, while FH is the main regulator of the AP, these interactions most likely are relevant during episodes of inflammation and/or tissue injury.

**Table 1:** Diseases related to factor H mutations or variants that have potential effects on interactions with CRP, PTX3, C3b or polyanions.

**Disease**	**Factor H or FHR polymorphisms/mutations**	**Interactions affected**	**Functional effect of disease-related variant**	**References**
AMD	FH Y402H (domain 7) FHL-1	CRP, polyanions	Insufficient clearance of drusen, inflammation	(46–50)
aHUS	FH mutations in domains 19-20	PTX3, C3b/d, sialic acid	C attack against vascular and blood cells, C-mediated inflammation	(24, 27, 51)
Atherosclerosis	FH I62V (associated with high MMP-8 levels)	C3b	Increased release of MMP-8 from neutrophils	(52)
DDD	FH domains 1-5 (e.g., R83S)	C3b	AP overactivation in the fluid phase, C3b deposition on basement membranes	(51, 53)
	FH Y402H (domain 7)	CRP, polyanions	Inflammation	(26)
C3GN	FHR abnormalities (e.g., hybrids), FHR5	CRP, C3b	Competition with factor H, AP dysregulation	(21)
	FH Y402H (domain 7)	CRP	Inflammation	(26)

*AMD, age-related macular degeneration; aHUS, atypical hemolytic-uremic syndrome; DDD, dense deposit disease; C3GN, C3 glomerulonephritis; FHR, factor H-related protein; AP, alternative pathway*.

### Age-Related Macular Degeneration (AMD)

AMD is a progressive blinding disease that makes the individual unable to perform basic activities requiring vision, such as reading, recognizing faces, and driving. Globally, AMD affects 170 million people. Therefore, it is the leading cause of visual disability in the industrialized countries. While age is the strongest risk factor for AMD, several genetic risk factors have also been reported. Of these, theY402H polymorphism in FH is the strongest (6).

**Figure 5:**
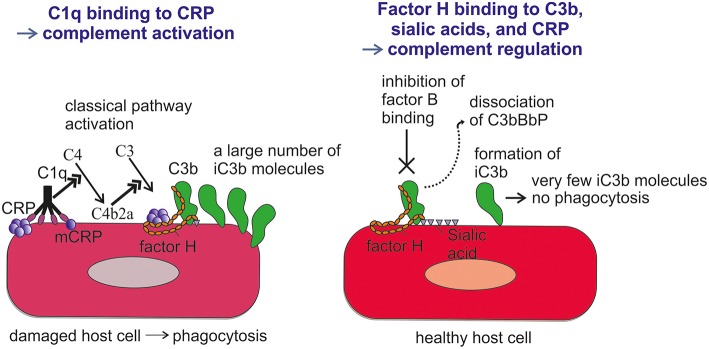
Role of factor H-CRP interaction in regulating complement activation on host cells during the course of inflammation. CRP deposition initially activates the classical pathway of complement on injured cells, which leads to deposition of large amounts of C3b. Binding of factor H to CRP blocks further AP activation and inflammation on the damaged cells by promoting inactivation of the C3b molecules to iC3b. In contrast, only a few iC3b molecules are formed on healthy cells due to the rapid inactivation accidentally bound C3b-molecules by sialic acid-bound factor H. On injured cells the density of iC3b molecules is high enough to lead to phagocytosis.

FH binds CRP at three sites, one located at domain 7, the second within domains 8 to 11 (32) and the third in domains 19-20 (57). CRP is thought to play an important role in helping to direct CP activation and suppressing AP activation at the site of tissue damage and during local inflammation. While CRP induces CP activation and C3b formation on apoptotic and damaged cells by recruiting C1q, the binding of FH to CRP blocks further AP activation and inflammation caused by accelerated C attack (Figure 5). Therefore, blocking of AP at this stage is crucial to prevent excessive damage of autologous cells and tissues at the site of inflammation.

An aberrant complement regulation may contribute to the etiology of inflammatory diseases, as exemplified by the strong association of the FH Y402H polymorphism with AMD (46–48). As a result of a single nucleotide polymorphism that leads to the substitution of tyrosine in position 402 in domain 7 of FH by a histidine, the binding of FH to CRP is reduced (49). It has also been observed that the 402 polymorphism may affect FH binding to certain local polyanions in the retinal tissue (58). The reduced binding of FH to CRP and/or to polyanions could partially explain why individuals homozygous for 402H have an up to 10-fold increased risk for developing AMD than individuals homozygous for 402Y. This is supported by the finding that drusen, lesions developed in early AMD between the basal surface of the retinal pigmented epithelium (RPE) and Bruch's membrane, contain numerous proteins associated with the complement system, including the membrane attack complex (MAC) (59). This same study found that drusen contain proteins common to extracellular deposits associated with atherosclerosis, elastosis, amyloidosis and DDD. Thus, suggests partially shared pathogenetic mechanisms for these diseases. However, the results of studies analyzing associations of FH and CRP with these diseases are still controversial.

The FH Y402H polymorphism is strongly associated with AMD. However, it is still unclear how diminished CRP interaction with FH contributes to the disease development. Ultimately, the binding of FH to both CRP and PTX3 prevents further complement activation. In the case of AMD, the described effect on the molecular interaction between CRP and FH is logical and supported by the synergistic effects between 402H homozygosity, CRP expression and AMD (60, 61). No genetic association to AMD has been observed with FH family proteins FHR-4 and FHR-5, although they are known to interact with mCRP. In contrast, individuals with an FHR-3–FHR-1 deletion have a smaller risk for AMD (40, 62). Because the binding sites in the C-terminal domains of FH and FHR-1 are nearly identical, it is possible that the protective effect of FHR-3–FHR-1 deletion could be primarily caused by the FHR-1 deficiency. Because FHR-1 competes out FH it could actually promote, rather than inhibit, AP activation on CRP-coated necrotic cells, although contradictory results have also been reported (41, 63). In addition to Y402H in FH, the same polymorphism is found in FHL-1. It has been suggested that FHL-1 is a major regulator of complement in the retinal Bruch's membrane, as it can passively diffuse through the membrane, whereas the full-sized FH cannot (50). In addition, FHL-1 was reported to have slightly different binding properties to CRP and PTX3 than FH (64).

### Atypical Hemolytic Uremic Syndrome (aHUS)

Hemolytic uremic syndrome (HUS) is a disease characterized by thrombocytopenia, microangiopathic hemolytic anemia and acute renal failure. The more frequent, “typical” form of HUS is associated with infections caused by Shiga-like toxin-(verotoxin) producing bacteria, such as enterohemorrhagic *E. coli* (EHEC), while aHUS is usually linked to mutations in complement proteins (FH, factor I, membrane-cofactor protein/MCP, factor B, C3), thrombomodulin or to antibodies against FH. aHUS is characterized by severe endothelial and blood cell damage, which is caused by a dysregulated and misdirected complement attack. Endothelial injury can be simulated *ex vivo* by the patient serum also in cases, where no mutations or autoantibodies have been found. These observations indicate that dysregulation of the AP on the cell surfaces is the central event in aHUS pathology (65). An abnormal recognition of cell or platelet surface sialic acids or C3b by mutated FH is the key mechanism behind the FH-mutation associated aHUS (66).

Binding of C1q to PTX3 has previously been shown to have a dual role, enhancing or inhibitory, upon C function. This depends on whether PTX3 recruits C1q to fluid phase molecules or to cellular surfaces, such as bacteria or apoptotic cells (67). Binding of the C-terminal domains of FH or of FHR-1 to PTX3 has been shown to be affected by aHUS-associated mutations within domains 19-20 of FH and by autoantibodies against FH and FHR-1. These findings suggest that a reduced binding of FH/FHR-1 to PTX3 could also have a role in the enhanced local C-mediated inflammation and endothelial damage in aHUS (27). Genomic rearrangements resulting in the generation of hybrid genes between FH and FHR-1 or FHR-3 or deletions are not unusual. From these, some have been reported to associate with aHUS or C3G but their interactions with PTX3 have not yet been studied.

### Atherosclerosis

Atherosclerosis is a disease, where arterial walls lose their dynamic properties because of lipid accumulation. The arteries may become narrow and in later stages obstructed because of plaque formation. Total obstruction, because of e.g., of plaque rupture, may lead to a local infarction e.g., in the myocardial coronary arteries. Atherosclerosis is considered to be a multifactorial disease driven by inflammation. Somewhat elevated levels of CRP are related to the long-term risk of death from cardiac causes (68). CRP is known to bind to phosphocholine (PC) and cholesterol in modified LDL particles and colocalize with LDL in human atherosclerotic lesions (37, 69). It has been suggested that FH has a protective role in the development of atherosclerosis, as it binds to apolipoprotein E and thereby increases cholesterol efflux by macrophages (70, 71). A marker of atherosclerosis, elevated level of matrix metalloproteinase 8 (MMP-8), was also strongly linked to FH gene polymorphisms in a large unbiased population study (52).

Accumulation of lipids in the lesions caused by inefficient removal of modified LDL by macrophages has been recognized in both atherosclerosis and AMD. Interestingly, AMD and atherosclerosis partially share similar pathological and histological features (72). Complement dysregulation may play a role in the development and progression of both diseases. However, the results of studies investigating the link between CRP, FH Y402H polymorphism and atherosclerosis have yielded controversial results (60, 73). Studies showing that mCRP dissociated from pCRP mediates local proinflammatory effects suggest that mCRP is a proatherogenic factor. mCRP might thus contribute to the formation of atherosclerotic plaques and induce plaque rupture or destabilization (74). To what extent polymorphisms or binding properties of FH or FHL-1 could alter mCRP effector functions has not yet been elucidated.

### C3 Glomerulopathy (C3G)

Dense deposit disease (DDD, membranoproliferative glomerulonephritis type II) and C3 glomerulonephritis (C3GN) constitute a group of rare kidney diseases (C3G). The kidney histology in DDD is characterized by the presence of dense deposits in the glomerular basement membranes in electron microscopy. The deposits stain for complement C3/C3b, while immunoglobulins are absent. The fundamental cause of DDD is relatively well-understood. The disease is due to hypercatabolism of the alternative complement pathway in the fluid phase and C3b deposition to targets (basement membranes) that lack membrane regulators of complement, like CD46 or CD55. C3 glomerulonephritis, however, is less well-understood. It is characterized by C3 deposits in the absence of glomerular dense deposits and immunoglobulins, although they may be present in small amounts. In a proportion of cases C3G is associated with monoclonal gammopathy (17, 75, 76).

Mutations, allelic variants, sequence duplications and deletions within the *FH/FHR* gene cluster are known to associate with C3GN and DDD (26, 53, 77). They include the Y402H polymorphism in the CRP interacting domain 7 on FH. One significant SNP in FHR-5 associates strongly with a particular type of C3GN. This SNP is located in the FHR-5 domain 1 that is homologous to the short consensus repeat 6 of FH, which interacts with CRP. This is particularly interesting as this could affect the FHR-5-CRP interaction, and thereby influence complement activation and control in C3GN (26).

### Other Diseases

According to what has repeatedly been shown, interactions between pentraxins, and the C system play a crucial role in the development and regulation of inflammation. These interactions play an important role in handling tissue damage and priming it for clearance. Thus, they are involved also in conditions such as cancer and infectious diseases, where tissue damage and necrosis often occur. It has been suggested that FH expression levels could be increased in certain tumors, such as urinary bladder and skin tumors (78, 79). In humans, PTX3 expression is increased in different cancers, while in mice FH recruitment by PTX3 to C3b deposited on tumor cells has been shown to restrict the development of local inflammation. This indicates that PTX3-FH interaction could play a role in tumor-associated inflammation (80). In a few studies, genetic polymorphisms in FH/FHRs have been associated with microbial infections (81–84), but further studies will be necessary to define their real significance. Probably indicating its importance, CRP only shows polymorphism in the non-coding regions that could influence its expression levels. Reduced expression of CRP has been observed e.g., systemic lupus. No deficiencies in CRP have been observed.

## Concluding Remarks

After the first discovery of the interaction between FH and pentraxins (32), it is now widely accepted that these molecules together regulate the balance between C activation and inhibition. Biochemical, histological and genetic data clearly link these factors to various inflammatory diseases indicating that they participate in the development and progression of these diseases. There are several polymorphisms and mutations in the pentraxin interacting domains of the FH family proteins. Some of them alter pentraxin-FH interactions suggesting a role for these molecules in disease development. However, further work is needed to characterize the exact molecular mechanisms and roles of pentraxin-FH interactions in the initiation and progression of inflammation in these diseases.

## Author Contributions

All authors listed have made a substantial, direct and intellectual contribution to the work, and approved it for publication.

### Conflict of Interest Statement

The authors declare that the research was conducted in the absence of any commercial or financial relationships that could be construed as a potential conflict of interest.
